# The Dangers of Hairdressing

**Published:** 1897-07-31

**Authors:** 


VOL. XXII.—No. 566.] [JULY 31, 1897.

### THE DANGERS OF HAIRDRESSING.

THE melancholy death of Mrs. Samelsohn from the explosion of the vapour of some liquid which was being used as an application to her hair, even although the jury has not yet returned a verdict upon the case, is full of warnings which ladies, and even gentlemen, should not fail to take to heart. It seems probable, from the evidence already given, that the basis of the "wash" was what is called petroleum spirit, or, more commonly, "benzoline," a liquid which has of late years been applied to many purposes in the arts, and has therefore become an ordinary article of commerce. This spirit is highly volatile and highly inflammable, insomuch that a moderate quantity of it, exposed to the air in a room, will in a few minutes disappear entirely, filling the room with a vapour which will explode violently if unduly heated, or if brought into contact with a flame. To say that it is thus volatile is another way of saying that it dries with great rapidity, and, as it is a powerful solvent of every kind of grease, it is much used for many cleansing purposes. It is supposed to form the chief agent in the "nettoyage à sec," which of late years has been so much advertised and practised; and it is largely used by typists for cleansing their machines. When rubbed upon the hair, either in its pure state or disguised by the addition of scent or of colouring matter, it rapidly removes all grease and dirt, and does so without producing the temporary change in the texture of the hair which follows upon washing with soap and water. Hence its application has become common for both sexes in hairdressers' shops, and is often offered to customers under the name of a "dry shampoo." It is all very well as long as the conditions of safety are secured; but these may so easily be departed from that prudent people will be more likely to avoid the spirit than to accept an assurance that there is nothing present by contact with which it can be inflamed. It is a mere surmise, but the explanation of Mrs. Samelsohn's death may be that the gas stove in the room, although it had been extinguished as a precaution against accident, was still hot, and that the vapour was liberated in sufficient quantity to explode from contact with the heated portion. The only thing certain is that petroleum spirit furnishes a highly dangerous explosive vapour, and that, if its use as a hair wash be continued, accidents from it will be inevitable from time to time. It is said—we do not know with what accuracy—that the sad case to which we refer has greatly excited public curiosity with regard to the hair wash, and that large numbers of people have besieged the shop with a request that, save in the matter of an explosion, they themselves should be dealt with in a similar manner. It is perhaps worthy of note that petroleum spirit is the reputed basis or solvent employed in making a much advertised quack embrocation; and hence, as it is well established that embrocations owe their chief efficiency to the rubbing with which they are applied, and not to anything which they may contain, it may be prudent on the part of sprained or rheumatic people to take such precautions as may reduce within reasonable limits the danger of being burnt to death.

The deadly character of more than one compound used for the hair forms a curious feature of the history of toilet appliances. Hair dyes, especially such as are advertised to contain no lead, have been fruitful sources of lead poisoning; and it is an open secret that the death of a very popular lady of high rank, who was much regretted a few years ago, was brought about in this manner. She could not reconcile herself to becoming grey, and her husband had not only a rooted objection to the use of dyes, but also to the smell of the particular one which the lady in question was accustomed to employ. The result was that she had it thoroughly washed off every night before retiring to rest, and reapplied in considerable quantity every morning. Her symptoms were clearly traceable to lead poisoning when once the clue was furnished, but this was not done until too late, and they were not sufficiently characteristic to lead to detection at an earlier period. When lead is not an ingredient, hair dyes, generally speaking, may be presumed to be innocuous; but it will not be wise of the purchaser to rely upon anything but the certificate of a competent analyst, or to put the smallest faith in the statements of the advertisers of such preparations. A good deal of what is sold as "glycerine soap" contains no glycerine; and a retailer has lately been prosecuted because he supplied under the name of "arsenical" some toilet preparation which contained only a scarcely appreciable quantity of arsenic.

Conversely, the dyes that are warranted to contain no lead will often be found to contain a good deal; and an independent analysis affords the only safety. We cannot, in this matter, profess to feel much sympathy with ladies who seek to change the colour of their hair in obedience to the requirements of caprice or fashion; and, if they suffer for their folly, we should be inclined to say that they were rightly served. But, in the conditions of modern industry, hair dyes are practically necessary for large numbers of working men, who would find it difficult to obtain work if they were grey, the external signs of age being often accepted as signs also of senile incapacity, of which there may perhaps be no other evidence. It is a familiar experience with merchant skippers that their crews are all dark-haired men at starting; and that, after a few days at sea, a large proportion of them become grey.

